# Growth Phase Dependent Cell Shape of *Haloarcula*

**DOI:** 10.3390/microorganisms9020231

**Published:** 2021-01-22

**Authors:** Sabine Schwarzer, Marta Rodriguez-Franco, Hanna M. Oksanen, Tessa E. F. Quax

**Affiliations:** 1 Archaeal Virus-Host Interactions, Faculty of Biology, University of Freiburg, Schaenzlestrasse 1, 79104 Freiburg, Germany; sabine.schwarzer@biologie.uni-freiburg.de; 2 Cell Biology, Faculty of Biology, University of Freiburg, Schaenzlestrasse 1, 79104 Freiburg, Germany; marta.rodriguez@biologie.uni-freiburg.de; 3 Molecular and Integrative Biosciences Research Programme, Faculty of Biological and Environmental Sciences, University of Helsinki, Viikinkaari 9, 00014 Helsinki, Finland; hanna.oksanen@helsinki.fi

**Keywords:** *Haloarcula hispanica*, *Haloarcula californiae*, haloarchaea, pleomorphism, cell shape, motility, archaellum

## Abstract

Several haloarchaea are reported to be pleomorphic, while others exhibit remarkable shapes, such as squares. Recently, *Haloferax volcanii* was found to alter its morphology during growth. Cells are motile rods in early exponential phase, and immotile plates in stationary phase. It is unknown if this growth phase dependent cell shape alteration is a specific feature of *Hfx. volcanii*, or conserved amongst haloarchaea. Here, we studied the cell shape and motility of two haloarchaea species *Haloarcula hispanica* and *Haloarcula californiae.* With a combination of light and electron microscopy, we observed that both strains undergo a growth phase dependent morphological development, albeit in a slightly different fashion as *Hfx. volcanii*. For both *Haloarcula* strains, the cell size is changing throughout growth. Cell shape seems to be related with motility, as highly motile cells on semi-solid agar plates are predominantly rod-shaped. We conclude that the growth phase dependent cell morphology alteration might be a common feature amongst haloarchaea, and that cell shape is generally linked with a motile life style. The conservation of this phenomenon underscores the importance of studies of the molecular mechanisms regulating cell shape in archaea.

## 1. Introduction

Microbial cells display great morphological diversity with varying shapes and sizes. Several underlying mechanisms have been discovered determining shape in bacteria and archaea [1,2,3,4,5]. In some species, the shape is uniform in the whole population, while in others this can vary depending on several conditions. The ability of a prokaryotic cell to actively restructure their shape in response to environmental conditions is called morphological plasticity. Pleomorphism describes a phenomenon in which a cellular population adapts its morphology depending on environmental factors or on growth phase. 

Several bacterial species like *Helicobacter pylori*, *Legionella pneumophila*, *Deinococcus radiodurans*, and *Borrelia burgdorferi* are reported to display morphological plasticity depending on environmental conditions [6,7,8,9]. Amongst the Archaea, pleomorphism is especially common amongst species belonging to the halophilic *Euryarchaea*.

Cell morphologies of haloarchaea have been described to range from rods and plates to triangular or even square cells [10,11,12,13,14]. Additionally, the model haloarchaeon *Haloferax volcanii* (abbr. *Hfx volcanii*) has been reported to display different cell shapes, including rod-shaped and plate-shaped cells [12,15,16]. Recently, improved microscopy techniques for haloarchaea [17], have allowed to study their cell morphology in more detail. Several studies have contributed to mapping the factors that determine cell shape in *Hfx. volcanii*, which display morphological plasticity. They undergo a growth phase dependent transition from rod-shaped to plate-shaped cells [15,16,18,19]. In early-exponential phase (OD_600_ < 0.1), cells are predominantly rod-shaped. They slowly transit to a mixed culture of rods and plates in mid-exponential phase (OD_600_ 0.1–0.6), and eventually have a homogenous plate-shaped morphology in late stationary phase [16,19].

In addition to the growth phase, also the composition of the media significantly influences cell shape. Stable rod-formation is mainly observed in medium supplemented with casamino acids (essential amino acids and small peptides), optionally substituted with trace elements [16,18,19]. Maintenance of the rod-shaped morphology is dependent on CetZ in *Hfx. volcanii*, which is a tubulin like cytoskeleton protein [15]. On the other hand, stable plate shape formation partly relies on the peptide archaeaosortase (ArtA). ArtA is responsible for the lipid attachment of *Hfx. volcanii* surface exposed proteins, including the S-layer, which is the main cell wall component of *Hfx. volcanii* [20].

Interestingly, cell shape is linked with motility in *Hfx. volcanii* [15,21]. Rod-shaped cells are very motile, while the plate-shaped cells are generally non-motile [15,16]. Only the rod-shaped *Hfx. volcanii* cells display an archaellum, the archaeal motility structure, at their cell surface near the cell poles [16,22]. In contrast, the plate-shaped cells usually possess a remnant of the archaellum motor without the filaments [16]. In addition, chemosensory arrays that allow for directional movement, are absent from the plate-shaped cells in stationary phase [16,22]. An archaeal MinD homolog, named MinD4, seems to be involved in cellular positioning of archaella and chemosensory arrays in the rod-shaped cells of *Hfx. volcanii* [18].

The accumulation of recent studies on the model *Hfx. volcanii* has now allowed for the identification of several environmental and cellular factors contributing to the maintenance of its cell shape, which together provide a clear overview of the factors determining the morphological plasticity by growth phase dependent cell shape change. However, it is currently unclear if this phenomenon is a specific feature of *Haloferax*, or more common under other haloarchaea. Therefore, we have studied the cell morphologies of other members of the class *Halobacteria Haloarcula hispanica* and *Haloarcula californiae*. The genome of *Haloarcula. hispanica* (abbr. *Har. hispanica*) is available [23]. In contrast, *Har. californiae* (abbr. *Har. californiae*) was more recently isolated and its genome is not sequenced [24,25]. Both organisms are attractive models for studies on archaeal viruses, as they are hosts for many different archaeal viruses representing tailed, icosahedral, pleomorphic and spindle-shaped virus morphologies [26,27,28,29,30,31,32]. *Har. hispanica* was originally reported to form a heterogeneous population consisting of short, motile pleomorphic rods by a length of 0.5–1.0 µm [25,33]. On other occasions, a small proportion of *Har. hispanica* population was additionally observed as large, non-motile, coccoid cells of 2–3 µm in diameter and a low frequency of thick ‘cluster’ like cells [34]. The cell morphology of *Har. californiae* has not been studied previously.

In this study, we conducted a detailed analysis of the morphology of two *Haloarcula* species and show that both display morphological plasticity and undergo growth phase dependent cell shape change, albeit both in a different fashion. In addition, we find that there is likely a similar link between growth phase and motility, as has been observed in *Hfx. volcanii*.

## 2. Materials and Methods

### 2.1. Media and Growth Conditions

*Har. californiae* ATCC 33799 [35] and *Har. hispanica* [33] cells were cultured aerobically at 37, 42, or 45 °C under constant rotation at 120 rpm. They were grown in media prepared with 30% (*w*/*v*) salt water (SW) containing (per liter) 240 g NaCl, 30 g MgSO_4_ · 7H_2_O, 35 g MgCl_2_ · 6H_2_O, 7 g KCl, 80 mM Tris-HCl (pH 7.2) and 5 mM CaCl_2_ [36]. The 30% stock of SW was diluted to make the working media with either 18% (*w*/*v*) or 23% (*w*/*v*) SW. For growth in rich YPC (yeast, peptone, casamino acids) medium [37] SW was supplemented with 0.5% (*w*/*v*) yeast extract (Difco, Thermo Fisher Scientific, Basingstoke, Hampshire, UK), 0.1% (*w*/*v*) peptone (Oxoid, Thermo Fisher Scientific, Basingstoke, Hampshire, UK), and 0.1% (*w*/*v*) casamino acids (Difco). For growth in rich modified growth medium (MGM) [38], 0.5%, (*w*/*v*) yeast extract and 0.1% (*w*/*v*) peptone were added. For growth in selective CA (casamino acids) medium [37], casamino acids were added to the SW to a final concentration of 0.5% (*w*/*v*). CA medium modified with trace element solution (CAB) [15] was prepared in the same manner as CA medium with addition of 1/100 expanded trace element solution. The trace element solution contains (per 100 mL) 36 mg MnCl_2_·4 H_2_O, 44 mg ZnSO_4_·7H_2_O, 230 mg FeSO_4_·7H_2_O, 5 mg CuSO_4_·5H_2_O (filter sterilized). The medium was set to pH 7.2 adjusted with KOH. Growth in different media and temperatures was monitored for 7 days. The experiments were performed in triplicates (containing 3 biological replicates) for each medium, temperature, and salt condition.

### 2.2. Motility Assay on Semi-Solid Agar Plates

Motility assays were performed as described previously [16,21]. Semi-solid agar plates were prepared with YPC, MGM, CA, or CAB medium made of either 18 or 23% SW containing 0.3% (*w*/*v*) agar. Cell cultures were grown to an OD_600_ of 0.5 and about 10 µL of culture was spotted on a semi-solid agar plate for inoculation. The experiments were performed in at least triplicates (containing at least 2 biological replicates) per plate. The motility ring formed after cultivation of plates at 45 °C for 6 days was analyzed by scanning of plates and measurement of the diameter using Fiji/ImageJ [39]. To analyze statistically significant differences of motility rings formed on media prepared with different salinities a nonparametric Mann–Whitney–Wilcoxon test was performed.

### 2.3. Phase-Contrast Light Microscopy

Phase-contrast images for cell shape analysis were recorded for *Har. californiae* and *Har. hispanica* grown in CA medium containing 23% SW. For microscopy, cultures from different growth phases were diluted to an OD_600_ of 0.1 and 5 µL cell suspension was placed at the centre of an agarose pad (0.4% (*w*/*v*) agar, 18% SW) prepared on a glass slide. A coverslip was placed on top. Images were acquired at 100x magnification in the oil immersion Phase contrast mode (PH3) using an Axio Observer.Z1 inverted microscope (Carl Zeiss, Oberkochen, Germany) and processed to analyze cell shapes using the plugin MicrobeJ of Fiji/ImageJ.

Samples taken from motility rings were incubated for 1 h at 45 °C in the respective liquid media. Agar was removed by centrifugation at 2000 g. The cells in the supernatant were observed with phase-contrast microscopy to analyze the cell shape and swimming behaviour on agar pads or in microscopy dishes, respectively. For imaging of motile cells, 1.5 mL of growing culture diluted to an OD_600_ of 0.1 was pipetted in a round DF 0.17-mm microscopy dish (Bioptechs, PA, USA) and observed at 100× magnification in the phase contrast mode. Time-lapse imaging was performed at 45 °C with an Axio Observer.Z1 inverted microscope (Zeiss) equipped with a heated XL-5 2000 Incubator running VisiVIEW software.

### 2.4. Automated Image Analysis of Cell Shapes

Phase-contrast images were analyzed using Fiji/MicrobeJ [40,41]. The circularity of free cells was determined by automated calculation. The minimum cell size was 0.3 µm^2^ (1 pixel = 0.065 µm). Fragmented cells, cells that contained holes, or cells that merged together in bigger aggregates were excluded from analysis. Cell circularities and diameters were calculated individually for all examined optical densities and binned into ranges of 0.001–0.100, 0.101–0.200, 0.201–0.600, 0.601–1.000, and 1.001–1.900 for further processing and analysis. An unpaired t-test was performed to test for statistically significant differences in cell diameters. Cell circularities are shown as frequency distributions grouped in bins of 7 intervals from 0.4 to 1. A nonparametric Mann–Whitney–Wilcoxon test was performed to analyze significant differences in cell circularities at certain optical densities.

### 2.5. Transmission Electron Microscopy

*Har. californiae* and *Har. hispanica* cells were grown at 37 °C in CA medium prepared with 18% SW buffered with 10 mM HEPES (4-(2-hydroxyethyl)-1-piperazineethanesulfonic acid) (pH 7.0). Cells from early-exponential phase cells were concentrated 10–20-fold by centrifugation and resuspension in CA medium before being adsorbed to glow-discharged carbon-coated copper grids (Plano GmbH, Wetzlar, Germany) with Formvar films. The samples were washed three times in drops of sterile 2 M NaCl and subsequently stained for 15 s with sterile filtered 2% (*w*/*v*) uranyl acetate prepared in 2 M NaCl. Grids were examined using a Hitachi 7800 transmission electron microscope coupled to an EMSIS Xarosa (EMSIS GmbH, Muenster, Germany) camera or an Zeiss Leo 912 Omega with Dual Speed 2K-On-Axis charged-coupled device (CCD) camera TRS, Sharp-Eye (TRS Systems, Moorenweis, Germany).

## 3. Results

### 3.1. Haloarcula sp. undergo a Cell Shape Transition during Growth

We analyzed the effect of different growth conditions on the doubling times of *Har. californiae* and *Har. hispanica*. To establish optimal cultivation conditions of both strains, common halophilic growth media with different compositions were tested (Figures S1 and S2). Both strains grew actively under aerobic conditions and had comparable doubling times. Within the tested temperatures (37, 42, and 45 °C), the shortest doubling times were achieved when cells were grown at 42 °C. The salinity of the medium had no significant effect on the doubling times of both strains (Figures S1 and S2). The shortest doubling times were 5.5 h and 6 h for *Har. hispanica* and *Har. californiae*, respectively, when strains were grown in CAB media at 42 °C.

When grown at 37 °C, both strains exhibited prolonged lag-phases. However, as soon as the strains started their logarithmic growth phase, doubling times were relatively comparable with the higher temperatures: doubling times of 7 h (*Har. hispanica*) and 6 h (*Har. californiae*) in CAB media and 10 h (*Har. hispanica*) and 9.5 h (*Har. californiae*) in YPC medium. We did observe that colonies of both strains grew faster on solid medium prepared with 23% SW, than with 18% SW. This corresponds with the reported optimal salinities for *Har. hispanica* and *Har. californiae* growth [25,33]. 

**Figure 1:**
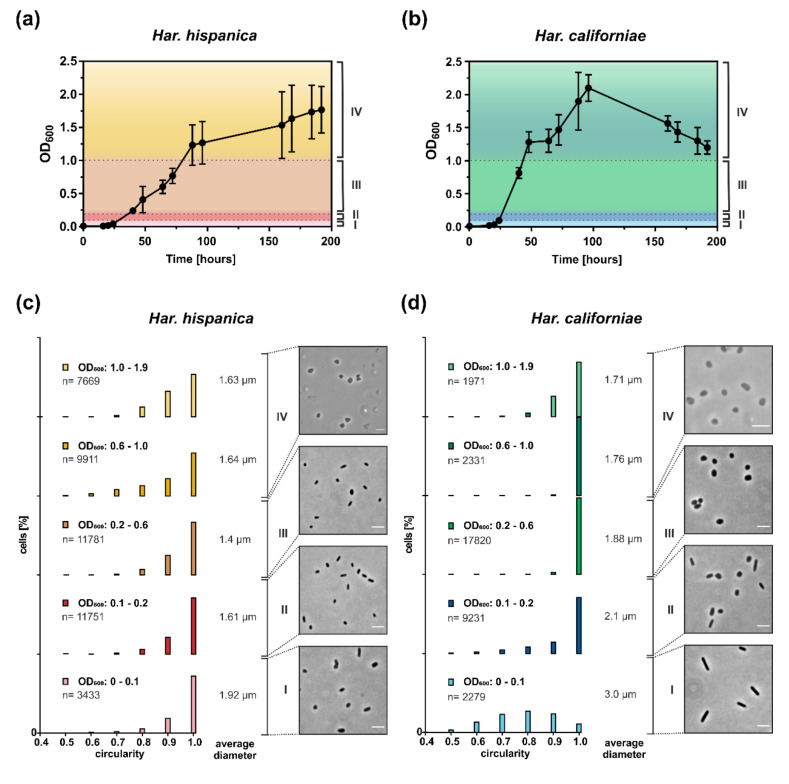
Correlation between growth phase and cell shape in *Haloarcula* sp. Representative growth curves of at least 3 independent biological replicates of (**a**) *Har. hispanica* and (**b**) *Har. californiae* grown in CA medium containing 23% (*w*/*v*) salinity at 42 °C. Curves represent mean of three technical replicates. Error bars represent the standard deviation of three technical replicates. Dotted lines indicate time points of sampling for cell shape analysis. For more biological replicates, see Figures S1 and S2. (**c**,**d**) The cell shape of *Haloarcula sp.* was analyzed using light microscopy. Representative phase contrast images of *Har. hispanica* and *Har. californiae* are shown from very early (I, OD_600_ 0.01–0.1), early (II, OD_600_ 0.1–0.2) mid (III, OD_600_ 0.2–0.1) and late (IV, OD_600_ 1.0–2.0) exponential growth phases. Scale bars represent 4 µm. (**c**,**d**) Relative frequency distributions of circularity measurements of (**c**) *Har. hispanica* cells and (**d**) *Har. californiae* cells from different optical densities (I-IV, see **a** and **b**). y-axis is percentage of cells. The sum of the bar heights per bin equals 100%. Nearly perfect round cells are displayed by a circularity of 1. Data binning was used to combine measurements from similar optical densities in intervals of 0.001–0.1, 0.101–0.2, 0.201–0.6, 0.601–1.0, 1.01–1.9. These intervals correspond to the four recognized growth phases of both *Haloarcula sp*. (see text). The only exception is that the late stationary phase is divided in two bins to better reveal addition shape alteration in this growth phase. Above each circularity graph, the OD_600_ of the bins is shown, as well as the total number of analyzed cells (n). The ‘average diameter’ displays the average cell diameter of the population in each bin.

As *Haloferax* cells have been shown to undergo a growth phase dependent shape change in CA medium [16,19], we also studied the cell shape of *Har. californiae* and *Har. hispanica* cells growing in CA (prepared with 23% SW) by phase contrast microscopy (Figure 1). We distinguish four growth phases: very early (I, OD_600_ 0.01–0.1), early (II, OD_600_ 0.1–0.2), mid (III, OD_600_ 0.2–0.1) exponential phase and late stationary phase (IV, OD_600_ 1.0–2.0). *Har. hispanica* cells were mainly observed as rod-shaped during most growth phases. In addition, some very short rods and round cells were also found during all growth phases. Only, in late stationary phase, some of the cells appeared slightly triangular (Figure 1c). This observation of cell shape is reflected by the relative cell circularity distributions of *Har. hispanica* cells from different optical densities (Figure 1c). Round cells typically have a circularity value of 0.9–1. During most growth phases, there is a significant population of *Har. hispanica* with a circularity <0.9. We detected a significant difference between each of the bins for each strain (*p* < 0.0001), apart from the two highest OD bins for *Har. hispanica* (0.6–1.0 vs. 1.0–1.9) and the 0.1–0.2 vs. 1.0–1.9 bin for *Har. californiae*. In early and mid-exponential growth phases, ~2% of *Har. hispanica* cells were extraordinarily large (~4–5 µm^2^) and pleomorphic in shape (Supplementary Figure S5a). Whereas the shape remained rod-shaped through most of the growth phases, the size of cells changed significantly. The average length of cells from early exponential was ~1.9 µm and this was slowly decreasing to ~1.4 µm in mid-exponential and 1.6 µm in late stationary growth phases (Figure 1c). The progressive reduction in the size of the cells is statistically significant between all bins (*p* < 0.0001). Thus, *Har. hispanica* also shows a growth phase dependent cell shape change, like *Hfx. volcanii*. However, it remains rod-shaped during almost all growth phases, and only transitions to slightly pleomorphic and triangular cells at very late stationary phase.

During early exponential growth (OD_600_ ~0.01–0.1), *Har. californiae* cells were observed as long rods (Figure 1d). In correspondence with the observation by light microscopy, the measured cell circularity of the population also revealed the high abundance of elongated rod-shaped cells in early exponential growth phase (until OD_600_ 0.1; Figure 1d). Only a few round cells (circularity 0.9–1) were observed within this population. Upon further growth, cells transitioned from rod-shaped to plate or round-shaped cells and formed a mixed population of the two cell shapes (Figure 1d). In mid-exponential phase (OD_600_ ~0.2–1), cells became increasingly plate or round-shaped. Just like *Har. hispanica*, some cells of *Har. californiae* from late stationary phases, appeared slightly triangular, represented by a higher frequency of cells with a circularity below 0.9 (Figure 1d).

Measurements of the cell length within populations of cells are consistent with the described growth phase dependent cell shape change. In early exponential phase, *Har. californiae* cells had a length of ~3 µm, and this decreased to ~1.7 µm in stationary phase (above OD_600_ 1.0) (Figure 1d). The decrease in cell size was statistically significant between all bins (*p* < 0.0001) throughout almost all optical densities. An exception to this are bins of OD 0.6–1.0 vs. 0.1–1.9. Similarly to *Har. hispanica*, we observed ~5% of large pleomorphic cells (diameter ~5–6 µm) at early log phase (Supplementary Figure S5b). This population decreased to 0.01% in mid-exponential phase.

The cell shape transition of *Har. californiae* is very reminiscent to the reported cell shape transition of *Hfx. volcanii* [15,16,19] and *Hfx. gibbonsii* [42], during growth in liquid medium. The exact ODs at which the transition from rod-shaped to plate-shaped cells occurs is species-dependent and different for all strains.

Comparison between the cell shape in CA medium prepared with 18 or 23% SW showed no difference between the media with different salinities (data not shown). Thus, both *Har. californiae* and *Har. hispanica* undergo a growth phase dependent cell shape change, which results in plate-shaped and triangular-shaped cells in late stationary phase. However, both species differ in the morphology of cells in early and mid-exponential phase.

### 3.2. Har. hispanica and Har. californiae Are Motile

As the cell morphologies of *Hfx. volcanii* and *Hfx. gibbonsii* LR2-5 are linked with motility [19,42], we studied if similar correlation can be found for *Haloarcula* species. Cells were grown in CA medium and observed at similar ODs as for cell-shape analysis with time lapse microscopy at 42 °C. In case of *Har. californiae*, no motile cells were observed at any of the analyzed ODs (Video 1). For *Har. hispanica*, we observed motility between OD_600_ of 0.1 and 1.5, where ~40% of the population showed swimming motility (Video 2). To test if the observed absence of motility for *Har. californiae*, could be dependent on the growth medium, time lapse microscopy was also applied to cells grown in YPC, MGM, and CAB media prepared with different salinity (18 or 23% SW). In all cases, no motile cells were observed (data not shown).

**Figure 2:**
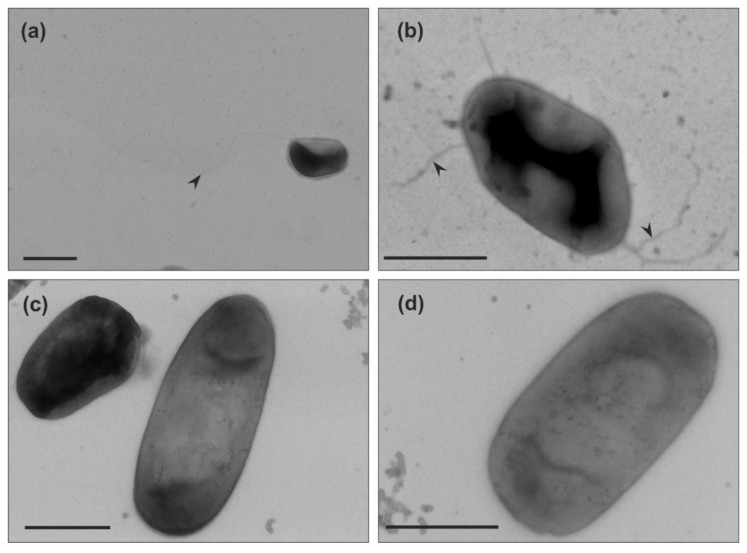
Transmission electron microscopy of (**a**,**b**) *Haloarcula hispanica* and (**c**,**d**) *Haloarcula californiae* morphology types from early-exponential growth phases (OD_600_ 0.1–0.2). Black arrow heads indicate the archaellum. Cells were negatively stained with 2% uranyl acetate. Scale bar, 1 µm.

After our analysis of motility in liquid medium of the two *Haloarcula* strains, we examined if archaella are produced in both strains. We harvested the cells at the mid-exponential growth phase (OD_600_ 0.1–0.2), where *Har. hispanica* is motile. Observation by transmission electron microscopy (TEM) showed that *Har. hispanica* cells usually contained one or two long archaella filaments at the cell poles (Figure 2a,b). In contrast, no cell appendages were observed on the surface of *Har. californiae* (Figure 2c,d).

**Figure 3:**
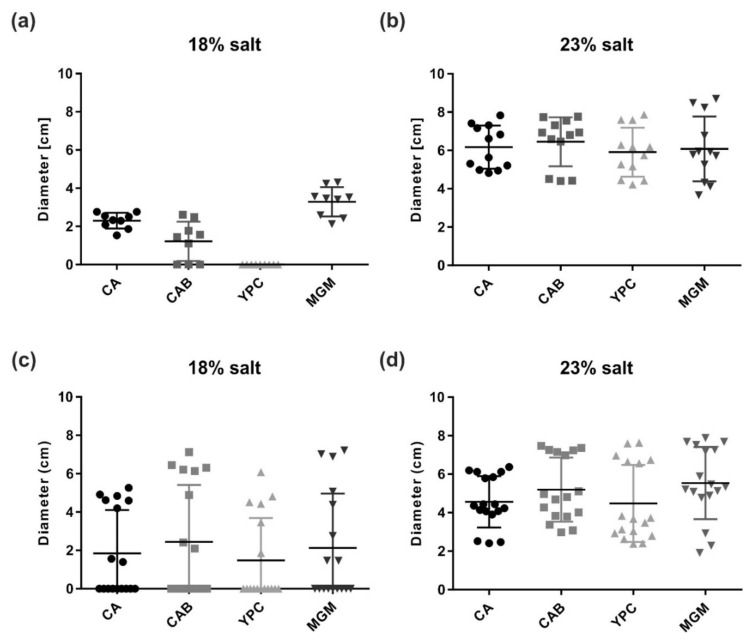
Motility of *Haloarcula sp*. on semi-solid agar plates. Quantification of the motility ring diameters formed by (**a**,**b**) *Har. hispanica* and (**c**,**d**) *Har. californiae* on semi-solid agar plates with different media in (**a**,**c**) 18% or (**b**,**d**) 23% SW. Calculations were made using more than three independent experiments including at least two biological replicates each. The middle black line indicates mean, lower and upper lines the standard deviation.

To test whether the motility of *Har. californiae* and *Har. hispanica* could be triggered in another fashion, cells were spotted on semi-solid agar plates. On semi-solid agar plates, cells have the possibility to swim away from the centre of inoculation, due to local starvation, and form a motility ring. By this assay, not only the functioning of the motility structure, but also that of the chemotaxis system is tested [43]. Motility assays revealed expanding motility rings of *Har. hispanica* and *Har. californiae* after 6 days of incubation (Figure 3). Salinity of the tested medium had a profound impact on motility, and the motility rings were always significantly larger in medium prepared with 23% SW for both *Har. californiae* and *Har. hispanica* than in lower salinity (*p* < 0.0001). Under higher salinity, the motility rings of *Har. californiae* and *Har. hispanica* had diameters of ~5 and ~6 cm, respectively. No motile cells of *Har. hispanica* were found when grown on YPC plates with 18% SW (Figure 3a). On CA, CAB, and MGM plates with 18% salinity, the motility rings of both strains were significantly reduced compared with those in similar medium with 23% SW. After 6 days, diameters were not more than ~2–4 cm (Figure 3 and Figure S3).

When the two strains were spotted on the same plate, exclusion zones between flanking motility rings were observed, suggesting that the cells can sense the presence of the other strain and stop motility in that direction (Figure S4a). This was also the case for motility rings produced by the same strain (Figure S4b). Similar findings were reported for *Hfx. volcanii*, where these exclusion zones also appear between motility rings of the same strain [15]. However, a mechanism by which cells could sense the leading edge of another motility ring, has so far not been discovered [15].

The above-described results indicate that both *Haloarcula* strains possess a functional chemotaxis system and motility structure. However, during growth in liquid medium, this motility seems not induced for *Har. californiae*. The absence of archaella at the surface of *Har. californiae* cells grown in liquid medium, suggests that the lack of motility observed under these conditions, is the result of downregulation of the archaellum filament production. Local starvation on the semi-solid agar plates might induce archaellum production and hence motility.

### 3.3. Cell Shape is Coupled With Motility on Semi-Solid Agar Plate

**Figure 4:**
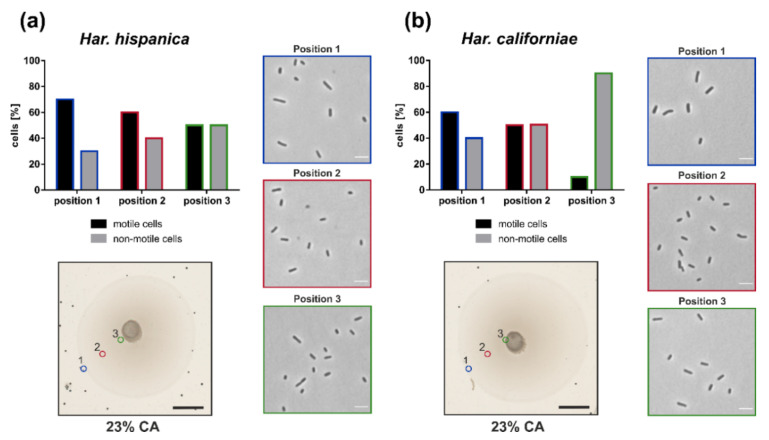
Cell shape and swimming behavior on semi-solid agar plates. Motility ring formation of (**a**) *Har. hispanica* and (**b**) *Har. californiae*. Photo in the lower left corner shows a typical motility ring on semi-solid agar plate, which indicated sampling positions 1–3 for cell shape analysis. Scale bars represent 1 cm. Images on the right, represent phase contrast light microscopy images of representative cell shapes at indicated sampling positions (colors from sampling positions correspond with coloring of the image frame). Scale bars represent 4 µm. Graph in the top left corner indicates the % of cells from the total population that is motile (black) or not motile (gray). Colors correspond with sampling positions from semi-solid agar plates.

After we observed that both *Har. californiae* and *Har. hispanica* formed motility rings on semi-solid agar plate, we analyzed the cell shape and motility of cells on semi-solid agar plates, to test for link between motility and cell shape. The motility rings were divided in three different zones, and with a pipet tip, cells were extracted from each zone. The cell shape and motility behavior of cells from each zone were observed by phase contrast microscopy. Cells obtained from semi-solid agar plates were predominantly rod-shaped and motile (Figure 4). Time-laps imaging revealed that upon resuspension in fresh liquid media and incubation at 45 °C, cells were actively swimming (Video 3,4). In all analyzed samples originating from motility rings, a mixture of motile rod-shaped cells and plate-shaped cells was observed (Figure 4). The majority of cells was rod-shaped and this cell morphology of the population was independent of the position on the motility ring from which cells originated.

Time lapse imaging of cells from the different zones in the motility ring showed different fractions of motile cells dependent on the sampling site (Figure 4). Whereas ~70% of *Har. hispanica* cells at the leading edge of the motility ring (Position 1) showed swimming behaviour, only ~50% were found to be actively swimming at the centre of the ring (Position 3). The amount of *Har. californiae* cells that were observed swimming when removed from motility plates, was lower compared to *Har. hispanica*. Only ~60% of *Har californiae* cells from Position 1 were actively swimming. This number decreased to ~10% when sampling at the centre of the motility ring (Position 3).

**Figure 5:**
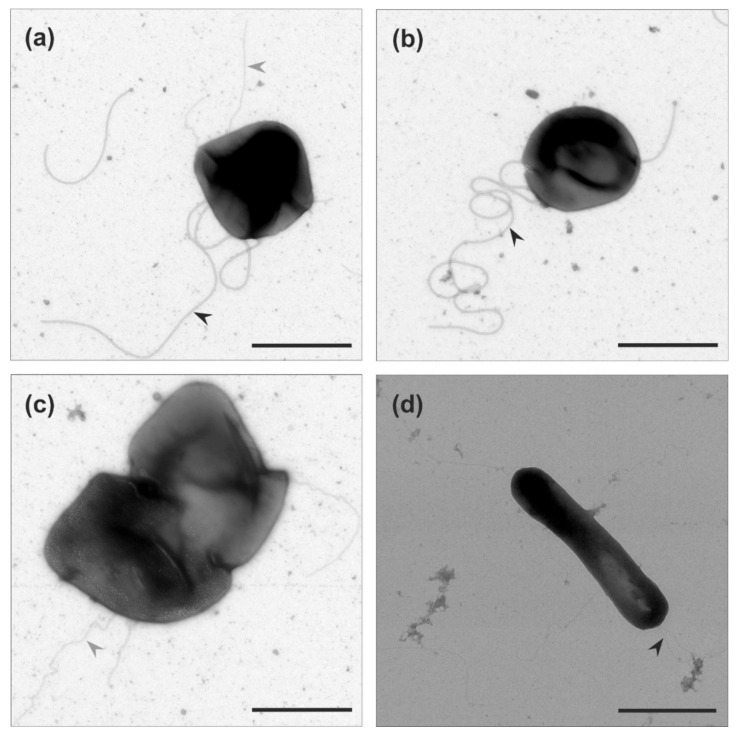
Transmission electron microscopy of (**a**–**c**) *Haloarcula californiae* cells with distinguishable archaella and pili and (**d**) *Haloarcula hispanica* rod-shaped morphology types from semi-solid agar plates. Black arrow heads indicate archaella. Grey arrow heads indicate pili. Scale bars represent 1 µm.

TEM of negatively stained cells from the leading edge of the motility ring showed that *Har. hispanica* and *Har. californiae* displayed filamentous structures at its surface (Figure 5). The majority were filaments of ~20 nm in diameter and 5–8 µm in length, that had a wavy appearance and often formed tufts. Therefore, they likely present archaella. The appearance of *Har. hispanica* was similar as those cells from liquid medium in mid-exponential phase (Figure 2a,b). In addition, on the surface of *Har. californiae* cells shorter thinner filaments (~11 nm) were occasionally observed as well, which we assume represent adhesive pili, as their diameter corresponds with such pili from *Hfx. volcanii* [44,45]. Archaella were observed both on rod-shaped and on round cells, although it is not sure that these observed cell shapes by TEM represent the natural cell morphology, as cells were not fixed during preparation.

## 4. Discussion

Most haloarchaea have initially been reported to display pleomorphic cell shapes. Recently, it was discovered that the cell shape of the model haloarchaeon *Hfx. volcanii* is dependent on the growth phase and it thus displays morphological plasticity [16,19]. Cells transit from rod-shaped to plate-shaped morphology in the course of early-exponential to stationary growth phases [16,19]. Moreover, cells shape was found to be linked with motility, and particularly the rod-shaped *Hfx. volcanii* cells are motile [15,16]. We now addressed the question if this growth phase dependent cell shape change is a specific characteristic of *Hfx. volcanii*, or if it is more common for haloarchaea. We studied the cell shape and motility behavior in detail during growth of two *Haloarcula* strains: *Har. hispanica* and *Har. californiae*.

Our results show that both strains undergo growth phase dependent cell shape alteration (Figure 1). In case of *Har. californiae* and *Har. hispanica*, the behavior is similar to that of that of *Hfx. volcanii* and *Hfx. gibbonsii* LR2-5 [15,16,19,42] as also a transition from rod- to plate-shaped cells was observed, although the transition took place later during growth for *Har. hispanica* compared to *Hfx. volcanii* and *Har. californiae* (Figure 1a,b). We observed three main differences between the *Haloarcula* and *Haloferax* species. (i) The presence of a small but constant population of extremely large pleomorphic cells in the early- and mid-exponential growth phase of *Har. hispanica*, which is not observed for *Haloferax*. *Har. californiae* cultures from early exponential phase also display a low fraction of extremely large cells, but these large cells disappear later during growth. *Har. hispanica* was originally described as containing 0.01–0.1% of large cells that are ‘cluster-shaped’ [26,34]. Such cells have been never observed for wild type *Hfx. volcanii* and their appearance is a bit like FtsZ knock-outs of *Hfx. volcanii* [46]. The morphology of these ‘cluster cells’ described previously for *Har. hispanica*, is similar to those that we observed for both strains in early and mid-exponential phase, as they contained ‘phase bright capsules’, and formed smaller clusters of divided cells (Figure S5). (ii) The second difference is that *Haloarcula* cells not only change their cell shape, but also generally become smaller. For both species, the average cell diameter is decreasing throughout development and cells become smaller towards late exponential and stationary phase. (iii) And finally, the *Haloarcula* strains generally contained a mixture of plate and triangular shaped cells in stationary phase, whereas *Haloferax* cells are mainly reported plate shaped in this phase, although triangular cells were also reported for *Hfx. volcanii* [11,19,42].

Interestingly, in *Haloarcula* species, the cell shape also seems to be linked with motility, as is the case for *Haloferax* [15,16,21]. Cells from motility plate are majorly rod-shaped. (Figure 4). However, the rod-shape on its own is not sufficient for motility, since we primarily observed non-motile rod-shaped *Har. californiae* cells in liquid medium in early-exponential phase. The reason for non-motile phenotypes of *Har. californiae* in liquid medium, is the lack of archaella in *Har. californiae* (Figure 2). This discrepancy between motility behavior in liquid medium vs. on semi-solid agar plates (Figure 5), can be explained by local starvation at the site of inoculation on the semi-solid agar plate, which functions as a trigger for the chemotaxis system, but might also indirectly induce archaellum production.

The observation that *Haloarcula* species, just like *Haloferax* species, undergo a shape change throughout growth, raise the question what the advantage of this morphological plasticity is. The link with motility might offer the most plausible explanation, as rod-shaped cells are likely more aerodynamic as plate-shaped cells. The early-exponential phase, where the cell density is low, might reflects environmental conditions where motility is advantageous for cells to move towards habitats with optimal growth conditions. The stationary growth phase with high cell densities resembles conditions immediately prior to biofilm formation, where motility and the coupled rod-shape appearance are no longer advantages. However, there are several other advantages that have been proposed for rod-shaped morphology in general; such as (i) improved fluid shear stress, which might be advantageous in environments with strong currents, or (ii) symmetry, which contributes to equal division over daughter cells [5,47,48]. The smaller cells size that was observed in densely growing cultures, might be advantageous because total surface area is increased and could be the result of selection due to nutrient limitation.

## 5. Conclusions

**Figure 6:**
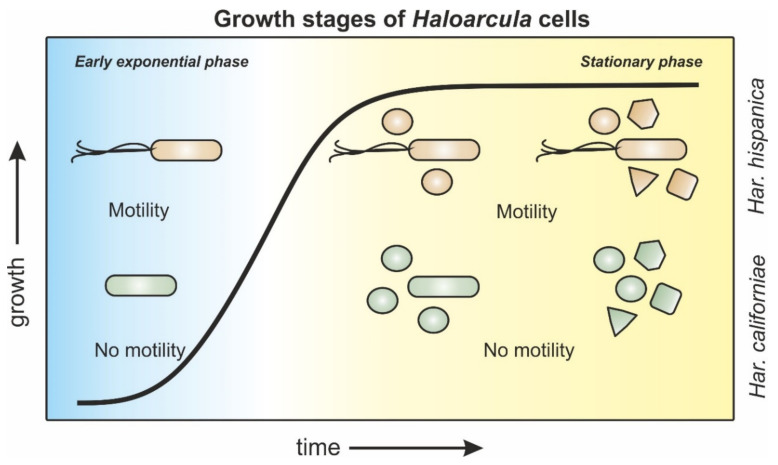
Schematic representation of morphology and motility behavior of *Haloarcula sp*. during different growth stages. *Har. hispanica* cells (orange) progress from rod-shaped, via a mixed population of rod- and round-shaped cells towards a late-stationary population with mixed cell shapes of rod-, round-, triangle-, and square-shaped cells. Rods are motile throughout all growth phases. *Har. californiae* cells (green) transition from rod-shaped cells in early exponential growth phases towards a mixed population of rod and round cells in mid-exponential phases. Cells from late exponential and stationary growth phases display rod-, round-, triangle-, and square-shapes. Cells are non-motile throughout all stages of growth.

The major findings of this work are summarized in Figure 6. These findings demonstrates that a growth phase dependent cell-shape change might be common amongst haloarchaea. Therefore, it underscores the importance of future studies on motility and cell-shape in haloarchaea to identify the main protein players, and the regulatory network in which they are connected. In addition, studies on other *Euryarchaea*, might answer the question if this trait is exclusive for haloarchaea, or more widespread.

## Data Availability

The data presented in this study are available in https://www.mdpi.com/2076-2607/9/2/231.

## Supplementary Materials

The following are available online at https://www.mdpi.com/2076-2607/9/2/231/s1, Figure S1. Growth analysis of *Haloarcula californiae* in different media and temperatures, Figure S2. Growth analysis of *Haloarcula hispanica* in different media and temperatures, Figure S3. Motility rings on semi-solid agar plates, Figure S4. Exclusion zones of motility structures of *Haloarcula hispanica* and *Haloarcula californiae*, Figure S5. Cell shapes of *Haloarcula sp.* deviating from the norm, Video S1: Time-laps imaging of non-motile cells of *Haloarcula californiae*, Video S2: Time-laps imaging of motile cells of *Haloarcula hispanica*, Video S3: Time-laps imaging of motile cells of *Haloarcula hispanica* from semi-solid agar plates, Video S4: Time-laps imaging of motile cells of *Haloarcula californiae* from semi-solid agar plates.
