# Supplementary material for: Growth Phase Dependent Cell Shape of Haloarcula
Source: Microorganisms. 2021 Jan 22;9(2):231. doi: 10.3390/microorganisms9020231 (PMC7911496; doi:10.3390/microorganisms9020231)
Supplement: Supplementary file 1 [file microorganisms-09-00231-s001.zip › 201218_Supplementary Information.pdf]

microorganisms

MDPI

Supplementary Information

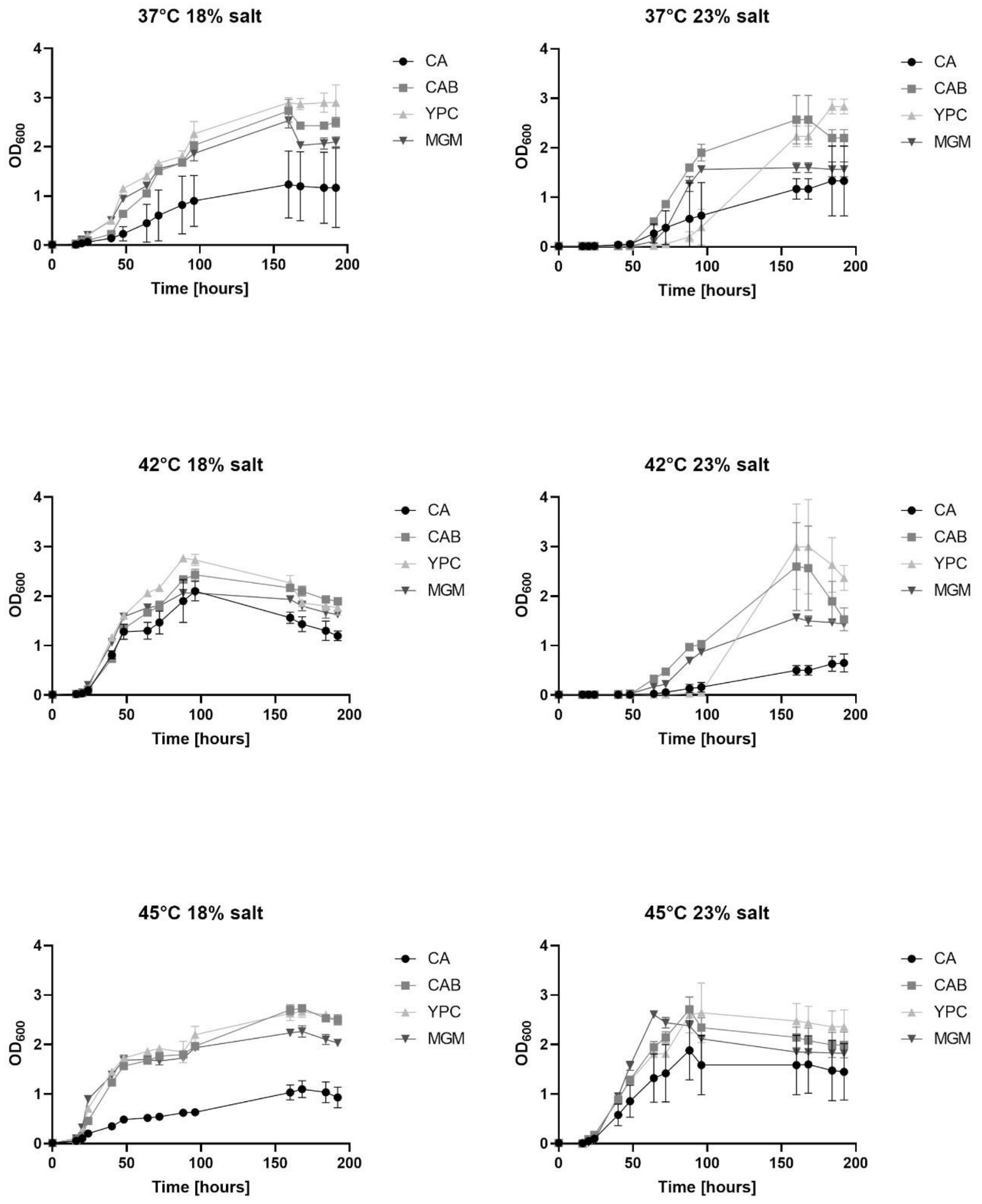

Figure S1. Growth analysis of *Haloarcula californiae* in different media and temperatures. Cells were grown in 18% [w/v] or 23% [w/v] salt concentration and complex nutrient media over a period of 192 hours. Figure represents one of three biological replicates with similar results. The average optical density at 600 nm (OD_{600}) was calculated from three independent technical replicates, error bars represent the standard deviation. On top of each graph the growth temperature and the salinity of the medium is indicated.

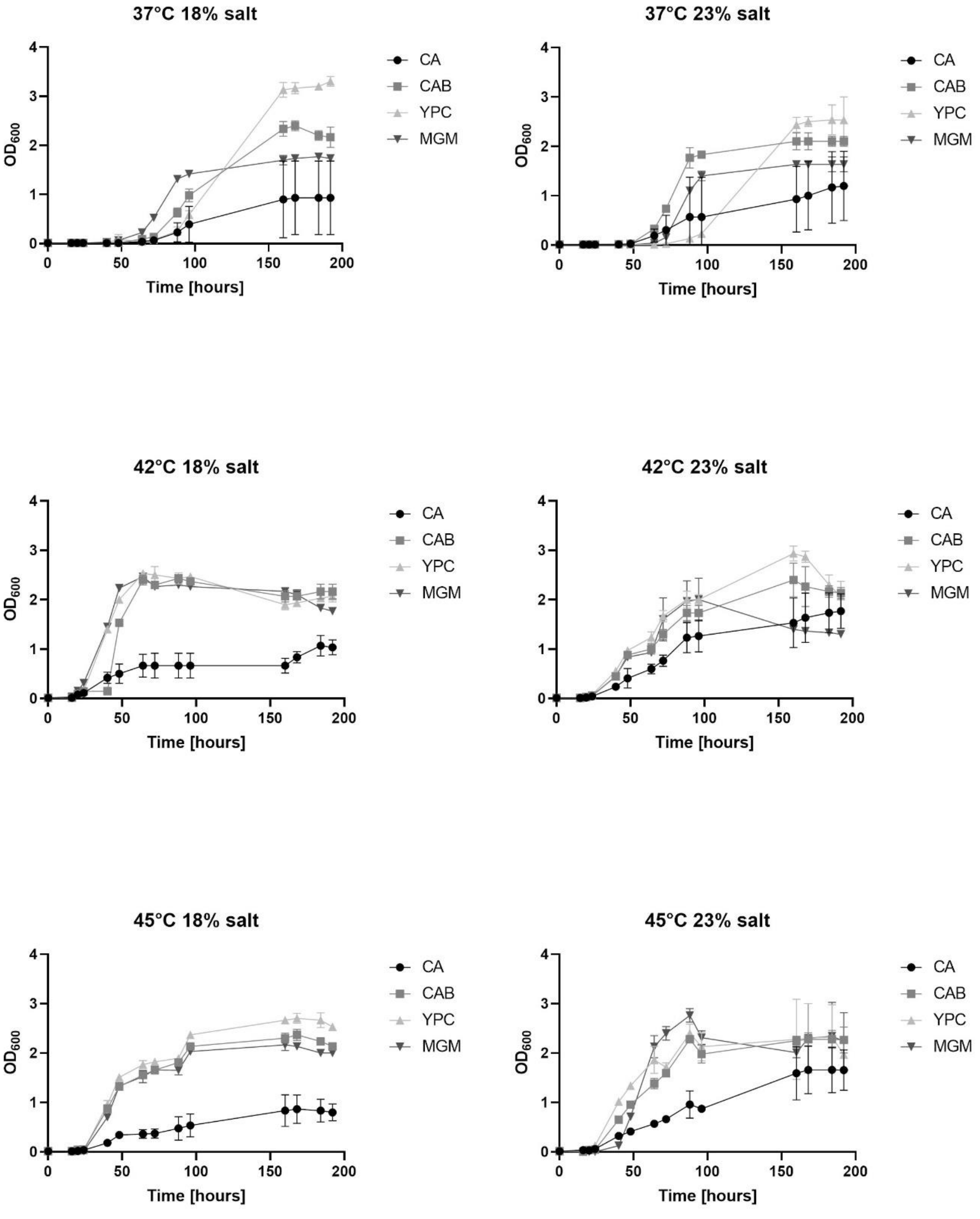

Figure S2. Growth analysis of *Haloarcula hispanica* in different media and temperatures. Cells were grown in 18% [w/v] or 23% [w/v] salt concentration and complex nutrient media over a period of 192 hours. Figure represents one of three biological replicates with similar results. Average optical density at 600 nm (OD_{600}) was calculated from three independent technical replicates, error bars represent the standard deviation. On top of each graph the growth temperature and the salinity of the medium is indicated.

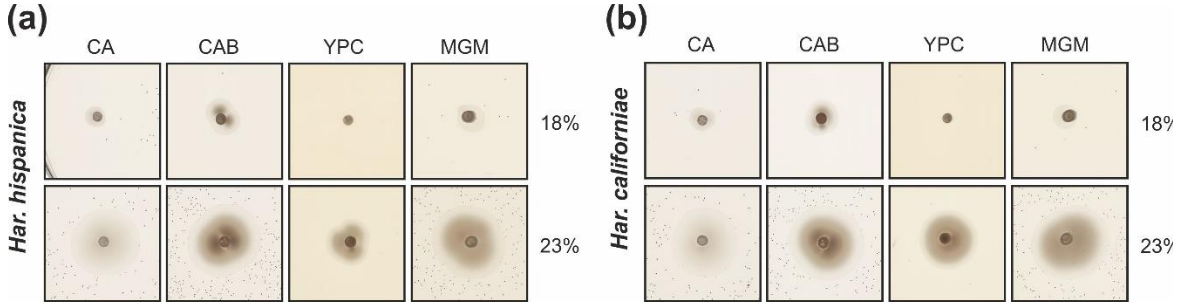

Figure S3. Motility rings on semi-solid agar plates. Representative example of motility rings from (a) *Har. hispanica* and (b) *Har. californiae* after 6 days of growth at 45 °C. Semi-solid agar plates were prepared with different media indicated on top containing 18% [w/v] or 23% [w/v] salinity.

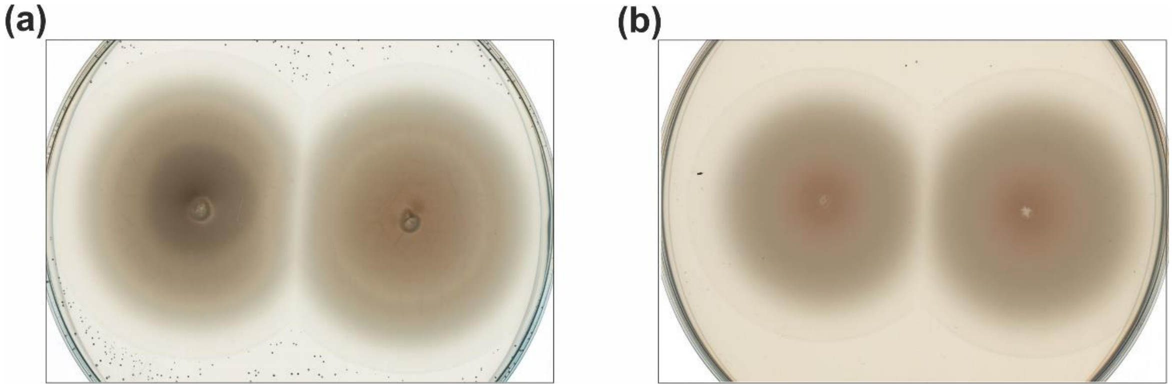

Figure S4. Exclusion zones of motility structures of *Haloarcula hispanica* and *Haloarcula californiae*. (a) *Har. hispanica* (left) and *Har. californiae* (right) on a semi-solid agar plate prepared with 23% SW and CAB medium. (b) Exclusion zones formed within two motility rings deriving from the same *Har. californiae* strain.

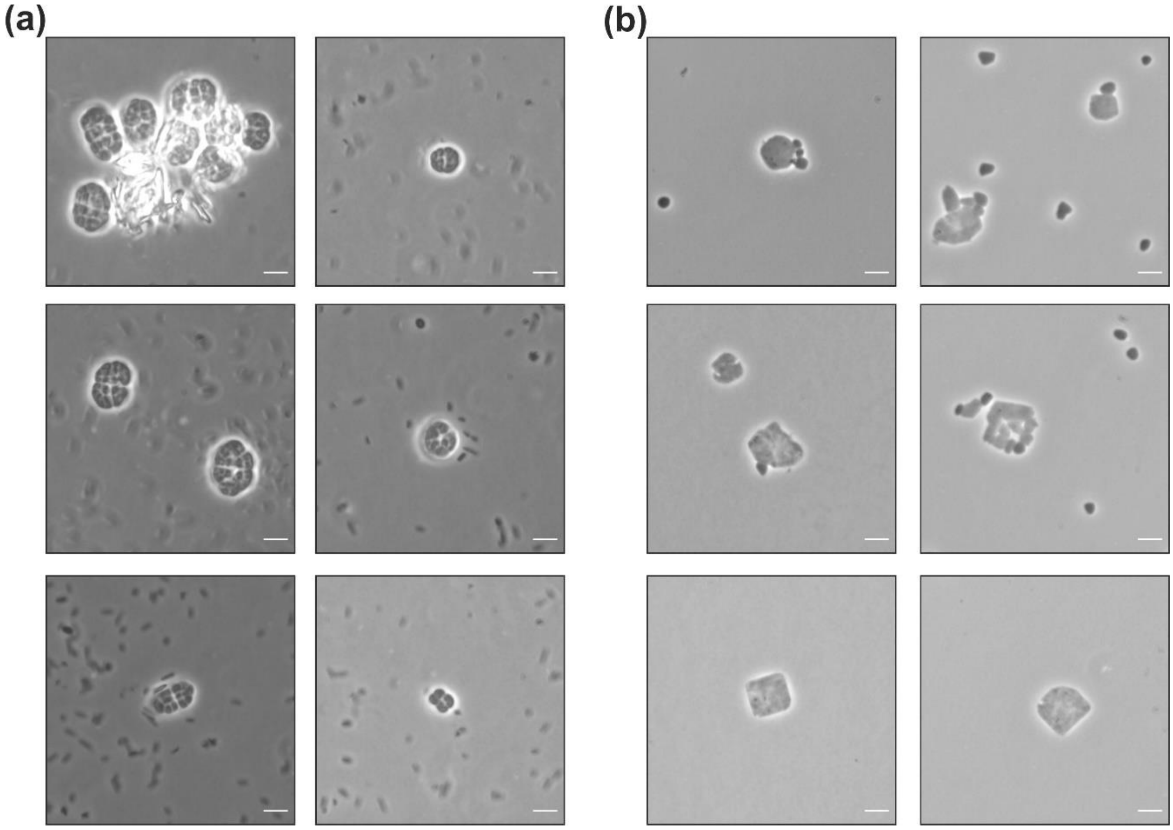

Figure S5. Cell shapes of *Haloarcula sp.* deviating from the norm. Different cell shape types of (a) – (f) *Har. hispanica* with average diameter of ~7.1 µm and (g) – (l) *Har. californiae* with average diameter of ~5.1 µm. Scale bars represent 4 µm. When large clusters and cells of *Har. hispanica* are focused, normal sized cells are automatically out of focus.

Supplementary Video descriptions

Video 1: Time-laps imaging of non-motile cells of *Haloarcula californiae*. Cells were prepared for time-laps-imaging after growth at 42°C in 23% (w/v) salt CA-medium.

Video 2: Time-laps imaging of motile cells of *Haloarcula hispanica*. Cells were prepared for time-laps-imaging after growth at 42°C in 23% (w/v) salt CA-medium.

Video 3: Time-laps imaging of motile cells of *Haloarcula hispanica* from semi-solid agar plates. Cells were withdrawn from the leading edge of a motility ring that was grown after 6 days of incubation at 45°C on a semi-solid agar plate prepared with 23% (w/v) salt CA-medium. C

Video 4: Time-laps imaging of motile cells of *Haloarcula californiae* from semi-solid agar plates. Cells were withdrawn from the leading edge of a motility ring that was grown after 6 days of incubation at 45°C on a semi-solid agar plate prepared with 23% (w/v) salt CA-medium.
